# Assessing anaerobic microbial degradation rates of crude light oil with reverse stable isotope labelling and community analysis

**DOI:** 10.3389/frmbi.2024.1324967

**Published:** 2024-03-07

**Authors:** Sebastian Beilig, Mark Pannekens, Lisa Voskuhl, Rainer U. Meckenstock

**Affiliations:** Department of Chemistry, Environmental Microbiology and Biotechnology (EMB) - Aquatic Microbiology, University of Duisburg-Essen, Essen, Germany

**Keywords:** sulfate reduction, community composition, oil reservoir microbiology, 16S rRNA amplicon gene, microbial community, oil microbiome, RSIL

## Abstract

Oil reservoirs represent extreme environments where anaerobic degradation profoundly influences oil composition and quality. Despite the common observation of biodegraded oil, the microbial degradation rates remain largely unknown. To address this knowledge gap, we conducted microcosm incubations with light oil as carbon source, original formation water and sulfate as electron acceptor, closely mimicking *in situ* conditions to assess oil degradation rates. Samples were taken from a newly drilled oil well to exclude contamination with injection water and allochthonous microorganisms. At the end of the incubations, microbial community analyses with 16S rRNA gene amplicon sequencing revealed the most prominent phyla as Desulfobacterota, Thermotogota, Bacteroidota, Bacillota (formerly Firmicutes), and Synergistota, collectively accounting for up to 44% of relative abundance. Ion chromatography and reverse stable isotope labeling were used to monitor sulfate reduction and CO_2_ evolution respectively. We calculated an average degradation rate of 0.35 mmol CO_2_ per year corresponding to 15.2 mmol CO_2_/mol CH_2(oil)_ per year. This resembles to approximately 200 years to degrade one gram of oil under the applied, presumably ideal conditions. Factoring in the available oil-water-contact (OWC) zone within the incubations yielded a degradation rate of 120 g CH_2_ m^−2^ OWC per year, closely aligning with the modeled degradation rates typically observed in oil reservoirs. Moreover, our study highlighted the utility of the reverse stable isotope labeling (RSIL) approach for measuring complex substrate degradation at minute rates.

## Introduction

Oil reservoirs are extreme habitats due to saturated hydrocarbon concentrations, low water activity and anoxic conditions (Pannekens et al., 2019). Nevertheless, microbial degradation is taking place in oil reservoirs where microorganisms favor the relatively easier-to-degrade light oil components such as alkanes over the more recalcitrant polycyclic aromatic hydrocarbons (PAHs) or asphaltenes (Larter et al., 2003; Mbadinga et al., 2011; Kayukova et al., 2016).

The microbial oil degradation alters the chemical composition of the oil in moderately temperate reservoirs leading to heavier oil or even bitumen with increased density (Head et al., 2003; Aitken et al., 2004; Youssef et al., 2009; Rajbongshi and Gogoi, 2021). Such processes take place over geological time scales, which makes it very challenging to assess the actual degradation processes and rates in oil reservoirs (Head et al., 2003; Larter et al., 2003; Aitken et al., 2004). Hence, microbial activities and rates resulting in hydrocarbon degradation in the subsurface remain still unknown. So far, there have been only a few studies that aimed at modeling *in situ* oil degradation rates at the oil water transition zone (Head et al., 2003; Larter et al., 2003) or directly determining the rate within a heavily degraded bitumen reservoir (Golby et al., 2012; Pannekens et al., 2021). Oil degradation rates certainly depend on several factors such as the reservoir temperature, oil column height, oil gravity (API) and chemical composition, diffusion coefficients in the porous media and maybe most importantly the oil-water distribution and interface area (Larter et al., 2006; Pannekens et al., 2019).

Biodegradation processes in oil reservoirs can be assessed by different methods such as oil-charging biodegradation models (Larter et al., 2003) or monitoring changes in the ratios of linear to branched alkanes (phytane or pristine) with gas chromatography – mass spectroscopy (GC-MS) (Kondyli and Schrader, 2019). Alternatively, the overall chemical composition of the crude oil can be analyzed through Fourier transform ion cyclotron mass spectrometry (FT-ICR-MS) analyses (Meckenstock et al., 2014; Cho et al., 2015). These qualitative measurements allow to assess the degradation state of oil samples from reservoirs but quantitative assessment of degradation rates is very difficult. Measurements of degradation rates have mostly been performed with laboratory incubations of oil samples where methane production or sulfate depletion were measured (Gieg et al., 2010). Methane can be very easily and sensitively measured with GC-analysis but sulfate measurements are often not accurate enough to monitor the minute metabolic rates that occur in anaerobic oil degradation. Alternative methods are thus needed to assess degradation rates of hydrocarbons in microcosm experiments.

We recently introduced the monitoring of hydrocarbon mineralization to carbon dioxide with reverse stable isotope labeling (RSIL) in order to measure the degradation of complex oil samples and obtain degradation rates (Schulte et al., 2019; Pannekens et al., 2021) which was not possible with classical methods such as analyzing oil compositions. For RSIL microcosms are set up with a bicarbonate buffer containing 10 atom percent of labeled H^13^CO_3_^-^. Upon degradation of hydrocarbons, carbon dioxide with natural abundance of ^13^C (1.01%) is released which dilutes the label in the bicarbonate buffer (Schulte et al., 2019; Pannekens et al., 2021). This change in the isotopic composition of the bicarbonate buffer can be very precisely measured with stable isotope analyzers for CO_2_. Pannekens et al. (2021) successfully applied this method to assess the anaerobic mineralization of natural bitumen. However, the monitoring took almost three years, which in many cases is too long for studying environmental processes.

With the present study, we intended at verifying if RSIL can be used to measure degradation of light oil and if the sensitivity and accuracy are sufficient for assessing degradation rates during shorter incubations. Furthermore, we wanted to determine potential degradation rates of a light oil reservoir using indigenous microbial communities from the formation water or water droplets entrapped in the oil and we compare the resulting rates with previously determined oil degradation rates of bitumen (Meckenstock et al., 2014; Pannekens et al., 2020, 2021). To this end, RSIL was applied to validate the biological activity and degradation rates of light oil in combination with anoxic formation water. Oil and formation water samples were gathered from a newly drilled oil well located off shore of Trinidad (Trinidad and Tobago) to prevent contamination with allochthonous microorganisms and incubated in microcosms amended with 10 atom% ^13^C-labeled bicarbonate buffer and sulfate as electron acceptor to follow the mineralization of oil to carbon dioxide.

## Materials and methods

### Oil sampling

Light oil and formation water from a newly drilled production well located in the Caribbean Sea close to Trinidad (Trinidad and Tobago) were acquired on 16.08.2018. Until sampling, the well remained free from water injection water, ensuring that the sample remained uncontaminated by production or sea water. Drilling fluids were not accessible. In total, the sample contained 16 liters light oil and four liters formation water. The formation water (402 mM Cl^-^, 47.8 mM PO_4_^3-^, 38.2 µM Mg^2+^, 48.8 µM Ca^2+^) was decanted from the oil phase and transferred into glass bottles (2 L) under anoxic and sterile conditions. The head space was flushed with N_2_/CO_2_ (80/20) and sealed with butyl rubber stoppers (Ochs Laborbedarf, Bovenden, Germany).

The composition of the sampled oil was unknown. However, available data pertains to the eastern Venezuelan basin. An examination of formation water extracted from offshore oil fields, specifically Samaan and Poui, revealed the presence of short-chained aliphatic acids indicating a light oil reservoir (Fisher, 1987).

Geochemical formation water compositions depend on the oil reservoirs geology and environmental conditions. Dissolved inorganic carbon (DIC) and dissolved organic carbon (DOC) of the formation water were analyzed with a DOC/DIC-analyzer (TOC-L, Shimadzu, Duisburg) and revealed 505 mg DOC L^-1^ and 1373 mg DIC L^-1^. The amount of DOC in the studied area is similar to those found in southern California (6 - 2930 mg L^-1^) and three offshore reservoirs in the Eastern Venezuelan Basin (200 - 16514 mg L^-1^) (Fisher, 1987; Mcmahon et al., 2018). However, the concentrations of DIC are higher in our study compared to the Eastern Venezuelan Basin with 4-765 mg L^-1^.

For the reverse-isotope-labeling method it is important that the DIC in the formation water is lower than 10 mM. To remove the carbonate, 4 M phosphoric acid was added to the formation water until the pH reached 2.9 and the DIC evaporated as carbon dioxide, the acidic pH was maintained for 5 minutes. Then, the pH was directly adjusted to 7.5 with an anoxic NaOH solution (4 M) and the carbon dioxide-free formation water was used for microcosm setup. The phosphoric acid treatment also removed dissolved calcium by precipitation of CaPO_4_ which was separated from the water by decanting. The acid treatment might have influenced the microbial community to a certain extent and may have led to a reduction in the observed initial activities. However, indigenous microbiota in subsurface oil reservoirs can be adapted to different pH conditions (Gao et al., 2016). Additionally, various mechanisms of acidic stress resistance (active proton pumps, cell internal buffer systems, changes in the membrane, chaperones) are known that might have protected the organisms from acidification resulting in the surviving community in our microcosms.

### Microcosm setup

Microbial incubations were set up in serum bottles (120 mL) adding one drop (0.33 ± 0.09 g) of light oil to each bottle. Afterwards, 96 mL of anoxic, carbon dioxide-free formation water was added and the serum bottles were flushed with N_2_/CO_2_ (80/20) and sealed with butyl rubber stoppers (Ochs Laborbedarf, Bovenden, Germany). The water was buffered with ^13^C-labeled bicarbonate (final concentration 10 mM with 10 atom % ^13^C) made from a mixture of ^13^C-labeled NaHCO_3_ with an ^13^C-atom percent x(^13^C) = 98% (Sigma-Aldrich, MO, USA) and regular NaHCO_3_ x(^13^C) = 1.11% (Carl Roth, Germany). Finally, the terminal electron acceptor sulfate (20 mM) and Na_2_S as reducing agent (0.5 mM) were added to remove any remaining traces of oxygen (Widdel and Pfennig, 1981). Immediate sulfate reduction proved that the bottles were all anoxic. Six replicate microcosms were prepared, whereof two were autoclaved to serve as negative heat-treated controls. The microcosms were incubated at 30°C in the dark in order to promote a wide range of microorganism without temperature restrictions. The overall duration of the experiment spanned 857 days, with monthly samplings conducted for the initial 122 days, followed by the final sampling point at day 857.

### Assessing light oil mineralization

To assess degradation rates of light oil by carbon dioxide evolution, 1 mL of the aqueous phase of each microcosm was sampled with a N_2_-flushed syringe through the stopper. The samples were transferred into 12 mL Labco Exertainer vials (Labco Limited, UK) that contained 50 μL of 85% phosphoric acid, closed with butyl rubber septa and screw caps, and flushed with carbon dioxide-free synthetic air (6.0 grade; Air Liquide, Germany) (Assayag et al., 2006; Dong et al., 2017, 2019; Schulte et al., 2019). The samples were analyzed using a Delta Ray CO_2_ Isotope Ratio Infrared Spectrometer (Thermo Fisher Scientific, MA, USA) with Universal Reference Interface Connect according to Pannekens et al. (2021). To achieve the highest precision possible, the carbon dioxide concentration of the reference and sample gas entering the machine was set to 380 ppm.

After measuring each sample for 5 minutes, the resulting ^13^C/^12^C isotope ratio given in δ^13^C values were averaged and transferred into isotope-amount fraction (*x*(^13^C)_CO2_) (Equation 1, Coplen, 2011).


(1) $$isotope\;amount\;fraction\;\left(x\left({\;}^{13}\text{C}\right){\text{CO}}_{2}\right)=\frac{1}{1+\frac{1}{\left(Delta\;Value\;Sample+1\right)\times VPDB\;Standard\;Ratio}}$$

The isotope-amount fraction depicts the relative amount of ^13^C in the gas phase originating from the DIC of the aqueous phase evaporated through the acid. The VPDB Standard ratio depicts the stable isotope ratio of the international Vienna PeeDee Belemnite carbon isotope standard given as δ^13^C = 0.0111802.

The calculated isotope amount fraction was furthermore converted into atom percent by multiplying by 100 (atom percent = *x*(^13^C)_CO2_ × 100 [%]). This value represents the abundance of ^13^C atoms within the sample in percent and is used to finally calculate the produced carbon dioxide according to Equation 2.


(2) $$produced\;CO_{2}=\frac{total\;CO_{2}\;in\;buffer\left[mM\right]\;\times \;\left(atom\;percent\;CO_{2}\;-{\;}^{13}\text{C}\;content\;of\;buffer\;\left[\%\right]\right)}{\left({\;}^{13}\text{C}\;content\;in\;substrate\;\left[\%\right]-atom\;percent\;CO_{2}\right)}$$

All calculations were performed using the actual total carbon dioxide concentration of 7.58 mM in the buffer and a ^13^C content of 1.075% in the substrate oil (Pannekens et al., 2021).

The depletion of the terminal electron acceptor sulfate was monitored via ion chromatography (IC) as described in Voskuhl et al. (2021). The samples were pretreated by addition of KOH (0.1 M) and subsequent centrifugation to remove particles and heavy metals. Furthermore, a solid-phase-extraction using octadecyl-modified silica gel columns (Macherey-Nagel, Germany) was performed to remove organic compounds from the samples.

The growth of microorganisms in the aqueous phase was monitored via microscopic cell counting. Cells were stained with DAPI (4,6-diamidino-2-phenylindole) and counted via fluorescence microscopy (Zeiss AxioScope.A1, Oberkochen, Germany) according to Saby et al. (1997).

### Microbial community composition

**Figure 1:**
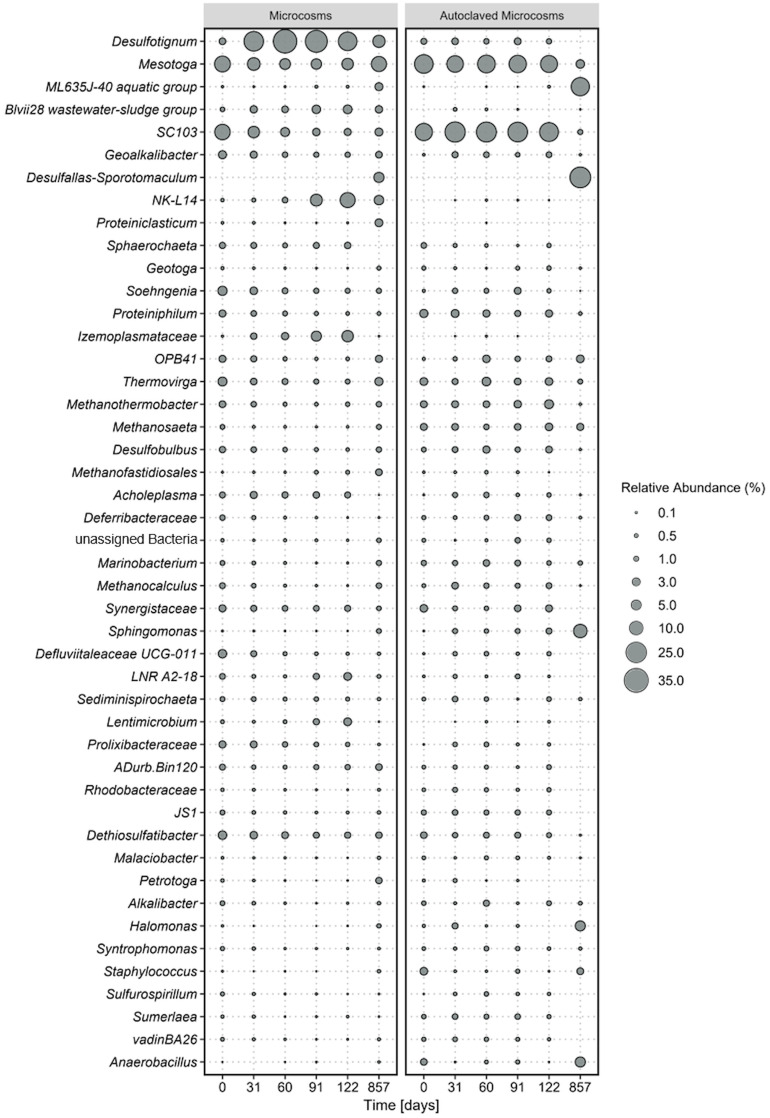
Average microbial community composition of most abundant taxa, calculated over four replicate microcosms (left panel) and two autoclaved controls (right panel) over 854 incubation days. Displayed taxa represent clustered ASVs on genus level. The size of each data point corresponds to the relative abundance in % at the corresponding time point.

DNA extraction was performed with 1 mL of aqueous sample from each timepoint and microcosm using the DNeasy PowerLyser Power Soil Kit (Qiagen GmbH, Germany) following the manufacturer’s instructions. Slight variations were conducted according to Pannekens et al. (2021). 16S rRNA gene amplicon sequencing of the V3-V4 region was performed using the primer set S-D-Arch-0519-a-S-15/S-D-Bact-0785-b-A-18 (Klindworth et al., 2013). A touchdown PCR was carried out to avoid non-specific amplification (Korbie and Mattick, 2008). The PCR products were purified (MagSi NGSprep Plus magnetic beads) and cleaned following the Illumina guide (“16S Metagenomic Sequencing Library Preparation,” 15044223). The last PCR step with Illumina indices and sequencing adapters was performed using the Nextera XT DNA Library Preparation Kit v2 Set D (FC-131-2004) by Illumina (München, Germany). After each DNA preparation step a 1% agarose-gel (w/v) electrophoresis was performed. The DNA concentration of each sample was normalized to 10 ng µL^-1^ and pooled into one ready-to-load library. Ready-to-load sequencing was outsourced to CeGaT GmbH (Tübingen, Germany) using a Illumnia MiSeq system with V2 500 cycle Flow Cell. Demultiplexing and adapter trimming was performed by the sequencing company. The resulting sequences were analyzed using the R-packages DADA2 version 1.24.0 and phyloseq version 1.40.0 (Mcmurdie and Holmes, 2013; Callahan et al., 2016). The forward and reverse reads were trimmed to 240 and 150 base pairs respectively before merging and chimera removal. Afterwards, the taxonomy was assigned using the SILVA database version 138.1 and the sequences were clustered into Amplicon Sequence Variants (ASVs) (Quast et al., 2013; Yilmaz et al., 2014; Callahan et al., 2016). In order to exclude rare organisms or sequencing errors, all amplicon sequence variants (ASVs) that either were only abundant in one of the two technical sequencing replicates or sequences with less than 10 reads per ASV were removed from the data set. Furthermore, all reads were rarified to the lowest detected read number of 14,212. Finally, the technical replicates of each timepoint were combined by calculating the mean of each ASV. Because many ASVs were received with the same taxonomy, we merged all of those with the “tax_glom” command always at the highest available taxonomic level, for visualization in (**Figure 1**). Raw sequences were deposited on the NCBI database (BioProject ID: PRJNA1030274).

## Results

### Mineralization of light oil

**Figure 2:**
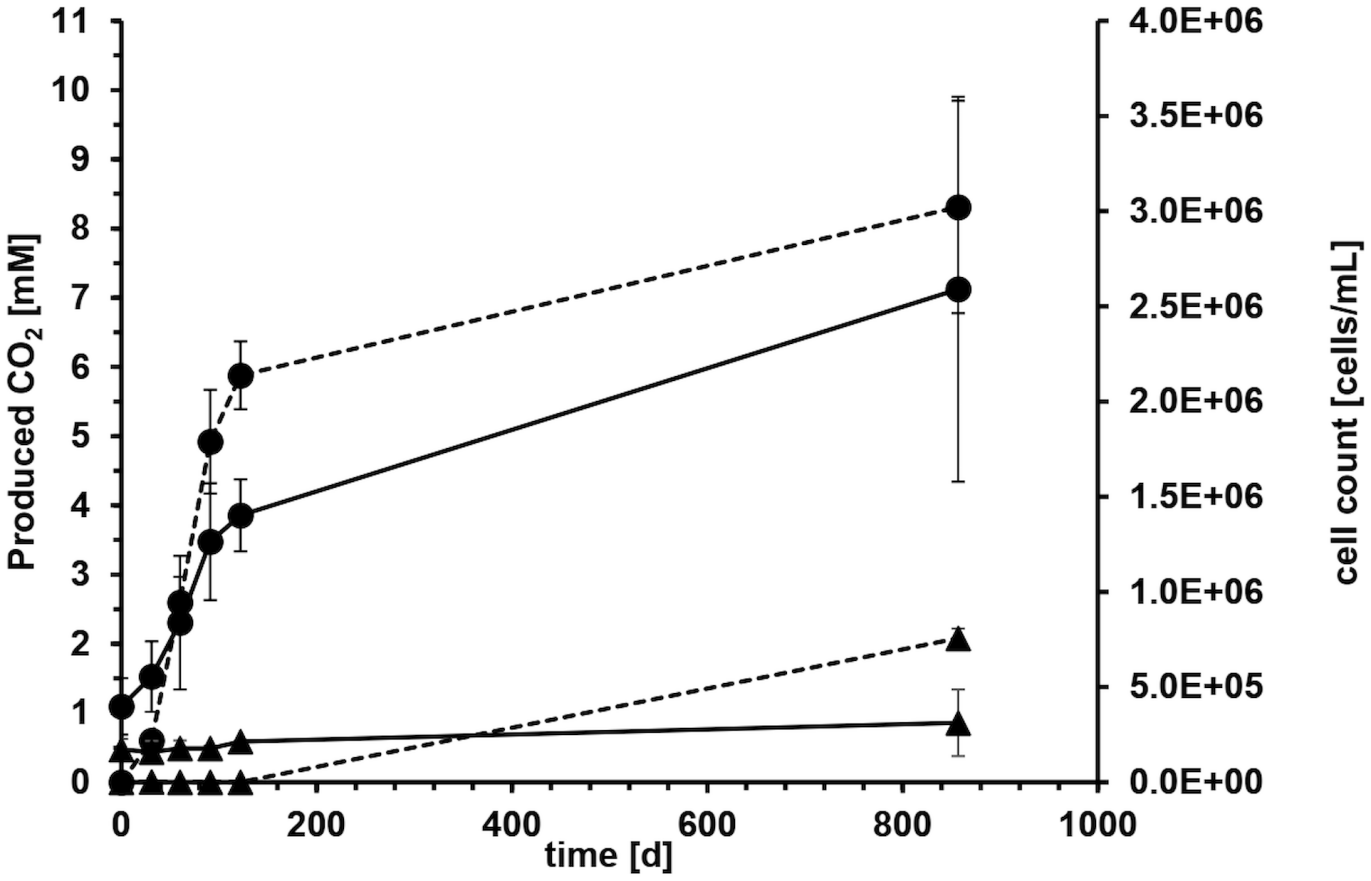
Average carbon dioxide evolution (dashed line) and cell counts (solid line) with crude oil as carbon and electron source and sulfate as electron acceptor of four microcosms over 857 days of incubation. Error bars depict standard deviations of four replicate incubations. Solid circles show life cultures (●) and solid triangles average values of heat-treated controls (▲) where the error bars indicate the spreading of duplicate incubations.

Microcosms with crude oil as carbon source and sulfate as electron acceptor were set up to determine biodegradation rates with reverse stable isotope labeling (RSIL) measuring the mineralization of oil to carbon dioxide. A major increase in carbon dioxide concentration was observed within the first 122 days of incubation with an average carbon dioxide production of 17.6 mM per year. The degradation rate in the four replicate microcosms was very reproducible within the first 122 days with 18.17 mM, 15.27 mM, 19.29 mM and 17.61 mM/year, respectively (**Figure 2**). The average production of carbon dioxide over the entire incubation period of 857 days was 8.31 ± 1.5 mM. Considering the volume of media used (100 mL), this resulted in the generation of 0.83 mmol of carbon dioxide after 857 days. Therefore, the overall degradation rate was 0.35 mmol CO_2_ per year or 1.06 mmol CO_2_ per gram of oil and year. This accounts to a rate of 15.2 mmol CO_2_/mol CH_2_(oil) per year or 120 g CH_2_ m^−2^ OWC per year. However, the majority of degradation activity occurred within the first 122 days of incubation with a degradation rate of 0.63 mmol CO_2_/mol CH_2_(oil) per day. The CO_2_ evolution demonstrated that 11.36 mg of oil were consumed throughout the entire incubation process. Given that we introduced 330 mg of oil, the electron and carbon source was not limiting.

The microorganisms in the system reduced the terminal electron acceptor sulfate, leading to a decrease from the initially added 20.6 mM to 15.2 mM over the 857-day incubation period indicating that the electron acceptor was also not limiting during the experiment. Considering the volume of media used, this corresponds to a total reduction of 0.54 mmol of sulfate and an average sulfate reduction rate of 0.23 mmol of sulfate per year.

However, the majority of sulfate degradation occurred within the first 122 days. Throughout the entire experiment, the pH values of the incubations remained stable at 7.5. The carbonate equilibrium at pH 7.5 implies that the majority of the carbon dioxide produced was completely dissolved as bicarbonate in the liquid phase, while a negligible amount was present in the gas phase.

An electron balance calculation was conducted based on the assumption that all the sulfate is reduced to hydrogen sulfide (5.4 mM). The crude oil used in our study is a complex blend of various hydrocarbons with an unknown oxidation state. Hence, we assumed a carbon oxidation state of -2 (CH_2_). The electron balance revealed that 88% (7.3 mM) of the electrons from the generated carbon dioxide (8.3 mM) were used for the reduction of sulfate. The remaining 12% are mostly likely used for biomass production which is a common yield value for sulfate-reducing organisms.

### Cell growth and community composition

Microbial growth in the aqueous phase was monitored during the exponential phase of carbon dioxide production. All microorganisms originated from the formation water or the oil itself. On average, the cell counts tripled from 4.0 x 10^5^ ± 1.5 x 10^5^ at the start of the experiment to 1.4 x 10^6^ ± 1.9 x 10^5^ cells mL^-1^ during the first 122 days (**Figure 2**). The cell counts in the autoclaved controls stayed mostly constant around 1.8 x 10^5^ ± 1.8 x 10^4^ cells mL^-1^ (**Figure 2**), probably because the cells were either dead or present as duration forms such as spores. High error bars at day 857 resulted from one autoclaved microcosm, where we could detect an increase in cell counts due to the proliferation of spore-forming microorganisms of the genus *Desulfotomaculum*. It remained unclear what triggered this germination and why it occurred in only one microcosm. The observed growth in the autoclaved controls can be attributed to several factors. Firstly, the autoclaving process was conducted in closed bottles to maintain anoxic conditions, necessary for the experiment. However, this method might have resulted in less efficient sterilization, as the microorganisms and water phase were not directly exposed to steam, leading to lower pressure compared to open bottles. Secondly, the closed system may have experienced reduced energy transfer and heating, potentially resulting in temperatures below the optimal 121°C during autoclaving.

Shifts in the microbial communities where analyzed based on 16S rRNA gene amplicon sequences of the V3-V4 region that have been agglomerated based on the genus level. Out of a total of 1926 amplicon sequence variants (ASVs), the most abundant taxa were selected for further analyses. Bacterial ASVs were more abundant (> 87%) than archaea (< 13%).

The most dominant phyla were Desulfobacterota, (*Desulfotignum, Geoalkalibacter, Desulfobulbus, SEEP-SRB1, Syntrophotalea*) (**Figure 1**), followed by Thermotogota, Bacteroidota, Bacillota (former Firmicutes), and Synergistota. ASVs of *Clostridiales* were also detected in our original oil microbial communities although not displayed in **Figure 1**, as individual abundances were all below < 0.2% at the start of the experiment. The five most abundant phyla account for 9-44% of the total relative abundance in the microcosms. Within the phylum Desulfobacterota, members of the genus *Desulfotignum*, *Geoakalibacter* and *Desulfobulbus* dominated the community composition in the first 122 days reaching in total a maximum of 58% relative abundance after 60 days. After the first 60 days the relative abundance of the three genera constantly declined to a minimum of 20% after 587 days. In addition, a rapid rise in members of the phylum Cloacimonadota and especially the genera *NK-L14* (Speth et al., 2022) and *LNR A2-18* (Dutra et al., 2023) were observed during this time. The relative abundance started at 2% and stayed stable within the first 60 days of incubation. After that, it rapidly grew to a maximum relative abundance of 17% after 122 days. This increase of members of Cloacimonadota happened simultaneously to the decline of members of Desulfobacterota.

At the start of the experiment, Thermotogota (*Mesotoga, SC103, Geotoga, Petrotoga, Defluviitoga, Kosmotoga, Pseudothermotoga, Oceanotoga, Thermotoga, Thermosipho*) was the most abundant phylum with a relative abundance of over 31%. However, the relative abundance constantly decreased in the first 91 days to a minimum of 9% while stabilizing around 20% at the end of the incubation. Most prominent representatives of the phylum Thermotogota belonged to the genus *Mesotoga* (Nesbo et al., 2012, 2019) and *SC103* (Gao et al., 2023). The microbial community composition in the autoclaved controls remained consistent throughout the entire incubation period, which correlated with the constant cell counts. At the final sampling point, the increased abundance of two spore-forming organisms in the controls suggested that autoclaving the closed, anoxic bottles did not completely eradicate all spores. Instead, it appears that some spores managed to germinate after 122 days of incubation.

After 857 days (2.3 years) the spore-forming genus *Desulfotomaculum* had probably proliferated and dominated the community composition of this control bottle. The growth of the spore-forming bacteria was mainly observed in the autoclaved controls and to lesser extent in the active microcosms.

## Discussion

Although oil reservoirs are extreme environments, microorganisms degrade the oil at the oil water contact (OWC) zone as well as in water phases within the oil leg (Head et al., 2003, 2010; Meckenstock et al., 2014; Pannekens et al., 2021). The objective of this study was to evaluate the potential of reverse stable isotope labeling (RSIL) to monitor small microbial oil degradation rates at the example of light oil from an oil reservoir located in Trinidad and Tobago. To this end, we collected reservoir fluids from a newly drilled production well including formation water and light oil without adding additional carbon sources. The oil well did not experience water injection ensuring that only the allochthonous microbiota was studied in the microcosm experiments. Using the RSIL, we were able to accurately and sensitively measure the rate of oil mineralization from a complex, naturally occurring light oil using the unaltered natural fluid composition.

In a previous publication, we conducted an extensive study on the degradation of heavy oil (bitumen) from the Pitch Lake in Trinidad and Tobago, which is a natural oil seep on the island of Trinidad (Pannekens et al., 2021). Since the incubation times in this experiment lasted over three years, we wanted to analyze here, if the RSIL method can be used to monitor the degradation of light oil in a reasonably shorter time frame. The bitumen degradation rate in the previous study was around 0.01 mmol produced carbon dioxide per gram of oil and year with a sulfate reduction rate of 0.46 mmol year^-1^ (Pannekens et al., 2021). The degradation of light oil in the present study was 1.06 mmol carbon dioxide per gram of oil and year with 0.23 mmol sulfate reduction per year. Thus, the reduction rate of light oil was 100-fold faster compared to the bitumen experiment (Pannekens et al., 2021). A conceivable explanation is that the short-chain hydrocarbons in the light oil are utilized easier than the extensively degraded bitumen.

Available data on anaerobic microbial degradation rates of crude oil are scarce. Numerical modeling of oil biodegradation at the OWC-zone have resulted in oil degradation rates of 10^−3^–10^−4^ kg petroleum m^−2^ OWC per year, dependent on reservoir temperature and oil composition. However, this rate was calculated from oil compositions based on gas chromatography measurements rather than from microbiological incubation experiments (Head et al., 2003; Larter et al., 2006). In order to draw a comparison between our findings and the modeled results, we normalized our determined degradation rate of 15.2 mmol CO_2_/mol CH_2_ per year to the oil-water contact-area of the incubations (16.61 cm^2^). The resulting degradation rate of 120 x 10^-3^ kg CH_2_ m^−2^ OWC per year closely aligned with the previous modeled rates of 10^−3^–10^−4^ kg petroleum m^−2^ OWC (Head et al., 2003; Larter et al., 2006). Nevertheless, one has to consider that the obtained rates in our study provide a degradation potential for the investigated sample under optimal incubation conditions and do not necessarily reflect real *in situ* degradation rates. Our microcosms provided very high concentrations of the favorable electron acceptor sulfate which is not present in pristine oil reservoirs and gets only introduced with injection water during secondary oil production. In oil reservoirs, mostly methanogenic degradation takes place which is orders of magnitude slower (Jones et al., 2008; Head et al., 2014). The microcosm incubations only contained an aqueous and an oil phase which diminished diffusion limitations that obviously occur in sediment or reservoir rock. Hence, the environmental conditions in the sediment or porous rock of the oil reservoir are distinct from those in the microcosm incubation. The obtained degradation rates primarily serve as an indicator of the presence and activity of microorganisms responsible for oil degradation in the original formation water and oil samples. Additionally, it is important that these degradation rates can be affected by various factors such as sampling of the environmental sample, storage conditions of the oil samples before incubation, and the experimental setup. For instance, the exposure to air before microcosm setup can rapidly lead to the demise of anaerobic microorganisms, potentially impacting the measured degradation rates. On the other hand, an exposure to oxygen will lead to an immediate growth of aerobic oil degraders, quickly changing the microbial community composition and the availability of easily degradable carbon sources. Nevertheless, our microcosm study can provide an estimate of the maximal degradation potential which provides a comparable parameter for biodegradation in different oil samples.

One way to qualitatively assess microbial degradation in oil reservoirs is by examining oil and gas samples for alterations in hydrocarbon composition, particularly the ratio of linear to branched alkanes. This analysis is primarily conducted to evaluate the weathering impact on crude oil and, to a lesser extent, to detect biodegradation, employing gas chromatography–mass spectrometry (GC–MS) techniques (Wang and Fingas, 1995; Munoz et al., 1997; Fernandez-Varela et al., 2009). However, this approach only offers qualitative insights into alterations in oil composition and does not provide information about degradation rates. Furthermore, it is conceivable that not all compounds from the intricate oil mixture are efficiently extracted during the sample preparation, which could result in their absence from the GC-MS spectra. This omission may potentially lead to overlooking degradation of non-identified compounds.

Since microbial degradation rates in oil reservoirs are extremely small, changes in oil composition or stable isotope ratios occur over geological time frames and cannot be monitored over time. Hence, such data have to be correlated to geochemical gradients or non-degraded reference samples to obtain indications of biodegradation but they can rarely provide degradation rates. However, gradients of compound compositions and nutrient supply capability have been used to model degradation rates in oil reservoirs (Larter et al., 2006; Kacewicz et al., 2010). There are two methods for assessing degradation rates through modeling. The first approach, known as the geochemical zero-order degradation model (referred to as “g0d”), entails the decomposition of hydrocarbons at a rate dependent on temperature. In this model, fresh oil containing known saturated hydrocarbon levels is introduced into a simulated geological trap at realistic rates (Larter et al., 2006). The second method, known as “Cyclops,” involves a coupled oil charging and biodegradation model with advective-diffusive mixing within the reservoir. This approach is employed to estimate degradation fluxes by analyzing compositional gradients in biodegraded oil columns consisting of multiple components (Larter et al., 2006). However, despite their cost and time efficiency, these modeling approaches suffer from limited accuracy due to their reliance on assumptions and simplifications that fail to fully capture the complexity of the entire system. This drawback is particularly pronounced when dealing with oil reservoirs. Nevertheless, the degradation rates derived from modeling aligned with the degradation rates observed in this study through experimentation.

Compound Specific Isotope Analysis (CSIA) is a good method to assess microbial degradation of oil, by measuring the carbon stable isotope composition of hydrocarbons (Rooney et al., 1998; Schmidt et al., 2004; Meckenstock et al., 2004a). The fundamental concept underlying this approach is that stable isotope effects occurring during biodegradation induce alterations in the stable isotope composition of specific compounds, which CSIA can effectively measure. It is essential to recognize that CSIA provides only a snapshot of the current situation. To gauge the extent of biodegradation, one can compare the stable isotope compositions of selected compounds with samples from non-degraded locations within the reservoir. Unfortunately, due to a lack of information regarding the duration of this degradation process, CSIA encounters significant challenges when attempting to calculate degradation rates in oil reservoirs.

An established method to assess the biodegradation of organic compounds like crude oil is to measure the increase in carbon dioxide partial pressure in microcosm (Struijs and Stoltenkamp, 1990; Battersby, 1997). However, this method is usually of limited sensitivity when small amounts of substrate are oxidized as in the case of anaerobic crude oil degradation. Since the measurement of the total amount of CO_2_ or the partial pressure is not very accurate, a small increase is hardly measurable. This problem can be overcome when microcosm incubations are amended with ^13^C or ^14^C-labeled substrates and the evolution of ^13^CO_2_ or ^14^CO_2_ is followed (Johnsen et al., 2013). Such measurements can be very accurate and sensitive but they are only applicable for specific labeled compounds that must be commercially available and cannot be used for complex mixtures or substrates of unknown composition such as crude oil.

To address this limitation, RSIL was developed as a robust and accurate method to assess the complete mineralization of all organic compounds in the sample (Dong et al., 2017). The advantage lies in accurately tracking the degradation of any organic substrate, including complex mixtures such as crude oil, by measuring changes in the isotopic composition of the carbon dioxide in the microcosm over time. In this study, we demonstrate that RSIL can serve as an excellent method for evaluating biodegradation rates of light oil from production wells or other environmental samples with complex and unknown substrate compositions.

Microorganisms inhabiting oil reservoirs have been identified as mesophilic and thermophilic sulfate-reducing, fermentative, manganese- and iron-reducing, acetogenic bacteria and methanogenic archaea (Magot et al., 2000; Li et al., 2017b; Pannekens et al., 2019; Zhou et al., 2019a, 2023). Comparing three oil seeps located thousands of kilometers apart revealed similarities in the most prominent representatives, which belonged to the bacterial phyla Proteobacteria, Bacteroidetes, Bacillota (former Firmicutes), Synergistetes, Deferribacteres, Thermotogae, Chloroflexi, and Bacteroidia (Pannekens et al., 2020). To this end, numerous systematic studies exploring the composition and distribution of microbial communities across water-flooded oil reservoirs have been documented. The dominant bacterial taxa encompass Alpha-, Beta-, Delta-, Gammaproteobacteria, Actinobacteria, Thermotogae and Sphingomonas (Feng et al., 2011; Wang et al., 2012; Gao et al., 2016; Li et al., 2017a; Kim et al., 2018).

It is obvious that the conditions within our microcosms differed from those in the oil reservoirs, as the microbial community compositions changed rapidly upon incubation (**Figure 1**). Examination of the microbial communities over time revealed the dominance the phyla Desulfobacterota, Cloacimonadota, Bacteroidota, Pseudomonadota, and Thermotogota. Interestingly, the abundance of these phyla did not remain constant over time. The primary energy conservation pathways during the first 122 days were most likely fermentation and sulfate reduction. Initially, the experiment started with a community that was dominated by the obligate anaerobic fermenters of the genus Thermotoga (Lanzilli et al., 2020; Shao et al., 2020), indicating that fermentation was the dominating metabolism in the original crude oil. Genomic analysis of Thermotoga indicated that they likely colonized oil reservoirs from the subsurface, rather than being originally deposited during oil reservoir formation (Nesbo et al., 2015, 2019). Following the incubation of oil, heavy oil, or water from oil reservoirs, fermenters frequently emerge as the most prevalent microorganisms (Mbadinga et al., 2011; Zhou et al., 2019b; Pannekens et al., 2021). In most studies on oil reservoir microbiology, sulfate reduction was observed to take a subordinate role due to rapid depletion of sulfate (Jones et al., 2008) or potential inhibition of the sulfate-reducing bacteria (Voskuhl et al., 2022). Typically when sulfate concentrations exceed 50 µM, sulfate reduction becomes the prevailing degradation process (Jimenez et al., 2016). The primary introduction of sulfate into oil reservoirs occurs through water injection. The allochthonus microbial community in our experiments was not exposed to injection water before because it was obtained from a newly drilled well. Nevertheless, the community was able to quickly utilize the added sulfate in our experiments which indicates that an oil reservoir community can rapidly produce reservoir souring upon sulfate injection.

Over the incubation time, Desulfobacterota took over and became dominant in the community within the first 60 days of incubation, probably due to reduction of the added sulfate. Then, the abundance of Desulfobacterota declined steadily over time, while the abundance of Cloacimonadota increased, suggesting a potential competition between these two phyla. Desulfobacterota, Thermotogota, and Cloacimonadota have been identified as core phyla specifically in low-temperature oil reservoirs (Hidalgo et al., 2021; Gittins et al., 2023; Zhou et al., 2023). Core phyla are phyla that are prevalent and most prominent within various oil reservoirs around the world. We could identify these phyla now in yet another oil reservoir confirming their status as core phyla.

The genus *Desulfotignum* (Desulfobacterota) can dominate sulfate-reducing communities from oil reservoirs containing high sulfate concentrations. This well-known member of oil reservoir consortia was very abundant in oil fields located in the Neuquen Basin (Argentina), Akita (Japan) and Jinangsu (China) (Grigoryan et al., 2008; Li et al., 2016; Li et al., 2017a; Kamarisima et al., 2018). Its prevalence in these environments can be attributed to its ability to utilize aliphatic organic acids, which are commonly present in petroleum reservoirs (Hatton and Hanor, 1984; Fisher, 1987, Means and Hubbard, 1987). In all anaerobic degradation pathways of aromatic hydrocarbons aliphatic acids are built as metabolites which can also be taken as indicators of microbial degradation (Gieg and Toth, 2020). The metabolites have to be unique and indicative of the degraded parent compounds. This is hardly possible for the majority of the alkanes in oil since the potential product fatty acids are universal to life. Only when specific modifications are performed, e. g. the activation with formate via glycyl radical enzymes, specific signature metabolites are produced that indicate certain metabolic pathways (Mbadinga et al., 2011). In the case of polycyclic aromatic hydrocarbons (PAHs) degradation, very specific signature metabolites are produced (e. g. different naphthoic acids) that can be attributed to the anaerobic degradation of naphthalene or other PAHs (Meckenstock et al., 2004b).

One interesting aspect of *Desulfotignum* is its ability to also degrade aromatics such as toluene through the fumarate-addition pathway (Hasegawa et al., 2014). Furthermore, its capacity to generate propionate from succinate and lactate further enhances its metabolic versatility and adaptability to varying reservoir conditions (Yang et al., 2017). The prevalence of *Desulfotignum* in moderate temperate oil reservoirs suggests a specific adaptation to such regimes (Tian et al., 2020).

Members of the Cloacimonadota have been identified in anoxic environments worldwide. Aside from engineered systems like anaerobic digesters (Dyksma and Gallert, 2019), Cloacimonadota have also been detected in natural environments such as sulfidic water layers of the Black Sea, Ursu Lake in Romania, and the Thuwal cold seep brine pool in the Red Sea (Williams et al., 2021). Based on incubations and analysis of Black Sea samples, Cloacimonadota have been inferred as fermentative heterotrophic generalists capable of utilizing various carbon sources, including proteins. As demonstrated here, they are also present in oil reservoirs as well as the biggest natural bitumen lake, the Pitch Lake in Trinidad-Tobago (Voskuhl et al., 2021). Furthermore, they were found in the high temperature Qinghai Oil Field, which was subject to water-flooding for more than 15 years (Hidalgo et al., 2021) or oil pipelines in Brazil (Dutra et al., 2023). Overall, the diversity of carbon utilization enzymes and metabolic pathways within the Cloacimonadota phylum suggests an anaerobic, acetogenic, and mixed fermentative lifestyle, potentially relying on flavin-based bifurcation of hydrogen during chemolithotrophic growth (Johnson and Hug, 2022).

Mesotoga are commonly found in mesothermic anaerobic environments rich in hydrocarbons. They are characterized by low or no hydrogen production as well as sulfur-compound reduction (Nesbo et al., 2019). Mesotoga are strictly anaerobic and their growth is slightly stimulated by thiosulfate, sulfite, and elemental sulfur (Nesbo et al., 2012). Sulfate reducing bacteria can potentially stimulate the growth of Mesotoga by sulfite production. Human activities, such as wastewater treatment and oil exploration, have expanded the habitats and dispersal of Mesotoga. It has been isolated from and detected in polluted marine sediments, low-temperature oil reservoirs, wastewater treatment plants, and anaerobic methanogenic environments, where it may participate in syntrophic acetate degradation (Nesbo et al., 2019).

Anthropogenic activities can significantly effect microbial communities in oil fields during production (Vigneron et al., 2017). In the initial stages of oil production, microbial communities were predominantly characterized by slow-growing anaerobes such as members of the *Thermotogales* and *Clostridiales*, presumably thriving on hydrocarbons and complex organic matter. During secondary oil production, the injection of seawater and nitrate induced significant alterations in microbial community composition with a shift towards fast-growing opportunistic species, encompassing members of the *Deferribacteres*, or Delta-, Epsilon-, and Gammaproteobacteria (Vigneron et al., 2017). These microorganisms exhibited a genomic profile indicating processes such as nitrate reduction and hydrogen sulfide (H_2_S) or sulfur oxidation. However, a change of the microbial community composition is not necessarily caused by injected microorganisms since the microbial communities in the oil reservoir do not necessarily contain allochthonous microorganisms (Mbow et al., 2024). Besides, findings from Gao et al. in 2016 elucidated specific microbial taxa consistently present in oil fields. Among others, members of *Geoakalibakter, Marinobacter, Sphingomonas*, and Thermotoga were identified in five out of nine analyzed oil fields in China (Gao et al., 2016). In our study, we detected the presence of several *Desulfobacterota* ASVs which was also found by Vigneron et al. in oil reservoirs before water injection. Hence, our study confirms the prevalence and persistence of these microbial groups across diverse oil field ecosystems.

Concluding, our results demonstrate that the reverse stable isotope labeling (RSIL) method provides a valuable method to assess the degradation of light oil by indigenous microorganisms. The found microorganisms and observed changes in microbial community composition over time show that fermentation could be a main metabolic trait in the investigated oil reservoir.

## Data availability statement

The datasets presented in this study can be found in online repositories. The sequence datasets generated for this study were deposited at NCBI under BioProject ID: PRJNA1030274.

## Author contributions

SB: Data curation, Formal analysis, Investigation, Methodology, Software, Validation, Visualization, Writing – original draft, Writing – review & editing. LV: Data curation, Investigation, Methodology, Software, Supervision, Validation, Visualization, Writing – original draft, Writing – review & editing. MP: Conceptualization, Formal analysis, Methodology, Writing – review & editing. RM: Conceptualization, Funding acquisition, Project administration, Resources, Supervision, Validation, Writing – original draft, Writing – review & editing.
